# Consensual Sexting among College Students: The Interplay of Coercion and Intimate Partner Aggression in Perceived Consequences of Sexting

**DOI:** 10.3390/ijerph17197141

**Published:** 2020-09-29

**Authors:** Tara L. Cornelius, Kathryn M. Bell, Tylor Kistler, Michelle Drouin

**Affiliations:** 1 Department of Psychology, Grand Valley State University, 1 Campus Drive, Allendale, MI 49401, USA; kistlert@mail.gvsu.edu; 2 Department of Psychology, Acadia University, Wolfville, NS B4P 2R6, Canada; kathryn.bell@acadiau.ca; 3 Department of Psychology, Purdue University-Fort Wayne, Fort Wayne, IN 46805, USA; drouinm@pfw.edu

**Keywords:** sexting, dating violence, sexual assault, coercion, intimate partner aggression

## Abstract

Recent empirical data suggests that the majority of adolescents and emerging adults utilize digital technology to engage with texting and social media on a daily basis, with many using these mediums to engage in sexting (sending sexual texts, pictures, or videos via digital mediums). While research in the last decade has disproportionately focused on the potential risk factors and negative consequences associated with sexting, the data are limited by failing to differentiate consensual from non-consensual sexting and account for potential influences of intimate partner aggression (IPA) and sexting coercion in these contexts. In the current study, we assessed the positive and negative consequences associated with sexting, using behavioral theory as a framework, to determine the relationship between an individual’s personal history of IPA victimization and the perceived consequences. Undergraduate students (*N* = 536) who reported consensual sexting completed a series of measures examining their most recent sexting experience, including perceived sexting consequences, and their history of sexting coercion and IPA. Results suggested that those reporting a history of any type of IPA victimization endorsed more negative reinforcing consequences after sending a sext, and those with a history of physical or sexual IPA victimization endorsed more punishing consequences after sending a sext than those without such history. Additionally, experience with IPA was found to be positively correlated with perceived pressure/coercion to send a sext. The implications of these data for research, policy, prevention, and intervention are explored.

## 1. Introduction

Given the ubiquitous nature of digital use among teens, adolescents, and emerging adults, it is perhaps unsurprising that technology is increasingly manifesting in their romantic and sexual relationships, including sending sexual texts, pictures or videos via digital mediums (sexting) [1,2,3]. Among emerging adults, smartphone ownership is estimated at approximately 95% [4,5], and data suggest that the vast majority are utilizing texting or social media on a daily basis [6,7]. Although early sexting prevalence estimates were widely variable based on methodological differences (2%–78%) [1,8,9,10,11], several reviews and current data suggest that sexting behavior among emerging adults is statistically normative, with current individual study estimates suggesting that more than 50% of college students have sent a sext and at least 60% had received a sext [3,12,13,14]. Recent meta-analytic data examining 50 studies and more than 18,000 emerging adults suggests slightly lower rates, with approximately 38% reporting sending a sext and 42% receiving a sext [15]. Additional findings indicate that roughly 48% of the combined samples reported reciprocal sexting, with publication date operating as a moderator such that more recent studies evidenced a higher prevalence of reciprocity of sexts [15]. These data align well with meta-analytic data on sexting behaviors among younger teens and adolescents, which suggest that both age and publication date were moderating factors associated with sexting, indicating that as individuals get older and as technology use has become more universal, sexting has increased [16]. The increase in availability of mobile devices, in tandem with the development of apps that reportedly enable private and convenient sharing of digital content (e.g., Snapchat), may have increased the accessibility and motivation for engaging in sexual behavior mediated through technology, including sexting, in the 10+ years since the advent of the smartphone.

Because sexting was initially largely conceptualized as a potentially risky behavior among emerging adults, much of the existing research has focused on the association between sexting and a range of risk factors, including unprotected sex, STI diagnosis, substance use, sexual coercion, pornography use, risky internet use, and psychological distress [1,3,17,18,19,20,21,22,23,24,25,26]. An additional area of concern related to sexting is the potential for coercion and aggression in interpersonal relationships that this behavior may afford. This is particularly important to consider given the high rates of intimate partner aggression (IPA) among emerging adults. IPA is a prevalent and serious public health concern that peaks in incidence among emerging adults, with current estimates suggesting that 20%–37% experienced physical aggression, and upwards of 70% experienced in-person psychological aggression [27,28,29]. Further, there are ample data to suggest that sexual coercion and aggression is common in this population, with current estimates suggesting approximately 13% of undergraduate students experience sexual victimization, with females being more likely to experience sexual aggression compared to males [30].

While several studies have examined aggression that occurs online among emerging adults (cyber dating abuse) [31], with some finding associations between the experience of cyberaggression and sexting [32,33], there has been only limited research examining the interactional relationship between in-person IPA and sexting behaviors. Morelli and colleagues [34] found that among moderate and high sexters, rates of IPA perpetration and victimization rates were higher, both in-person and online. There are also some data to suggest that sexting may be an additional mechanism through which sexual violence occurs. For example, in their sample of teens, Kernsmith, Victor, and Smith-Darden [35] examined the relationship between in-person sexual coercion and coercive sexting victimization and perpetration. Their data suggested that, compared to non-victims, victims of coercive sexting were at significantly higher risk of at least one form of in-person sexual coercion from their dating partner [35]. Similarly, Titchen and colleagues [36] demonstrated that teen sexting is associated with in-person sexual abuse in dating relationships, and other research involving incarcerated men has shown a relationship between sexting and sexual violence perpetration [37]. Data examining the interplay of IPA and sexting with emerging adults specifically suggested that IPA was significantly positively associated with sexting coercion [38]. Recently, Ross, Drouin, and Coupe [17] examined the relationship between different types of IPA and sexting coercion, particularly the cumulative effects of these experiences on mental health symptoms. The findings of this study suggested that individuals who experience IPA, sexual coercion, and sexting coercion in combination tend to experience the most severe psychological and sexual problems, compared to those who experience IPA alone. This suggests a pattern in which sexting coercion is presenting a cumulative risk, in combination with IPA, that contributes to increased psychological distress above and beyond the severity of the individual aggression experienced [17]. Although these data provide preliminary support suggesting a relationship between in-person IPA and sexting behavior, additional research is necessary to examine how in-person IPA impacts the experience and perceived consequences of sexting for emerging adults.

Research examining risk behaviors has predominated the literature in sexting; considerably less research has focused on the potential consequential benefits of sexting [39]. Some data suggest that individuals perceive sexting as a way to continue to connect in a relationship, particularly during college when partners may be separated [10,40,41]. Additionally, researchers have shown that sexting is associated with increased sexual satisfaction [42] and relationship satisfaction [40,43,44,45], though this may vary as a function of attachment style and gender. Recent latent profile analyses involving Americans and Canadians in cohabiting relationships [42], however, showed that nonsexters did not differ from those who sexted often (frequent and hypersexters) on relationship satisfaction, and sexters also fared worse in terms of other relationship markers, such as conflict, ambivalence, and attachment security. Other researchers have examined expectations and motivations for sexting and found that, particularly for men, emerging adults noted positive expectancies more often than negative expectancies and often cited motivations including flirtation, fun, sexual explorationand sexual initiation [10,42]. Among adults, Doring and Mohseni [46] found that, among those who had sexted, the perceived positive outcomes significantly outweighed perceived negative outcomes, and half of participants perceived no negative effects of sexting. Considerably less research has been conducted on the perceived consequences of sexting within the context of a particular relationship, which may be more illuminative in exploring the contexts in which positive outcomes are most likely to occur. Drouin, Coupe, and Temple [14] examined how relationship context (casual versus committed) may affect the perceived consequences of sexting. Although approximately 50% of the total sample reported positive or neutral outcomes related to sexting, this varied by gender and relationship type. In general, those in non-committed relationships and women reported fewer positive and more negative consequences than did those in committed relationships and men. However, this body of research is limited by the scope and depth of the positive consequences that were evaluated. For example, Doring and Mohseni [46] measured positive perceived outcomes through a general question (“How far has sexting had a positive impact on you?”), and Drouin, Coupe, and Temple [14] noted only emotional and sexual connection as possible “positive” consequences. This is a consistent limitation across a range of studies examining perceived positive outcomes of sexting [41]. As sexting becomes increasingly normative among this population, it is important to more systematically examine the range and context of potential desirable sequelae of sexting, as well as the various contextual and historical events that may contribute to the manifestation of sexting behavior, including IPA.

Several theoretical explanations have been offered to help understand human behavior generally, and coercive and aggressive behavior specifically. Bell and Naugle [47] developed an integrated, contextual model of IPA that drew heavily on existing research and behavioral theory, which postulates that the consequences of behavior are important predictors of future behavior. More specifically, behavior that results in reinforcing consequences is expected to be more likely to occur again in the future, whereas behavior that leads to punishing consequences is anticipated to occur less frequently in the future. Previous research examining IPA consequences has demonstrated that perpetration is often influenced by reinforcing consequences, which may explain, in part, the perpetuation and stability of aggressive behavior over time and across contexts [48,49]. There are also data to suggest that perpetrators of aggression, particularly dating aggression, are unlikely to experience punishment as a result of their behavior [49,50]. Of particular interest here is the role of negative reinforcement in maladaptive relationship behaviors, such that behaviors increase in the future, but do so by reducing negative affect, getting their partner to cease a behavior, stopping an argument, or assuaging guilt. These processes may simultaneously increase behavior, but represent potentially problematic relationship dynamics that should be addressed. To date, this theoretical model has not been applied to the understanding of coercive sexting, but the extant literature provides sufficient justification for its application beyond IPA.

While the initial research on sexting has been foundational to our understanding of this technology-mediated phenomenon, further research is necessary to understand how individual variables affect sexting behaviors. Critically, much of the initial research on sexting fails to differentiate between consensual and nonconsensual sexting behavior [1,10,11,12,14,20,38,51], or explore the nuances of consensual sexting. In a recent qualitative study, Roberts and Ravn [7] found that participants articulated the subtle, tricky, and sometimes unclear process of ensuring consent in sexting, and the ambiguity inherent in the practice. Further, online sexting guidelines in popular media sources often advocate for consent in sexting [52], and yet there has been little empirical research examining consensual sexting in particular. While the research on sexting is relatively nascent, it is critical to differentiate sexting behaviors that are coerced, unwanted, or nonconsensual from those that occur in consensual interactions. Even in a systematic review entitled, in part, “Consensual Sexting among Adolescents” [9], an analysis of the studies included and the nature of the questions does not necessarily indicate consensual sexting (e.g., “Have you ever sent a sexually suggestive nude or nearly nude photo or video of yourself to someone else?”) [11]. It is possible that the sending of this explicit material is not consensual, but could be influenced by partner pressure, coercion, or threats. Limited research has examined the role of unwanted but consensual sexting (i.e., willing engagement in technology-mediated sexual behavior that is unwanted). In one study with young adults [53], more than half of the sample indicated that they had engaged in unwanted sexting, and cited various motivations for doing so, including foreplay, flirtation, to fulfill the partner’s needs, and, for some, to avoid an argument. Other research has found that receiving unwanted sexts or sending consensual but unwanted sexts independently predicted a range of psychological symptoms, including depression, anxiety, stress, and lower self-esteem [54]. Recent research examining consensual, active sexting further demonstrated that even in this “consensual context”, approximately one third of participants reported pressure to sext, and sexting was consistently associated with poorer mental health outcomes for women specifically [55]. This further points to the need to continue to examine the psychological and coercive elements of sexting that is specifically identified as consensual.

In order to continue to advance the field, research needs to be conducted to determine the possible behavioral, interpersonal, and relationship consequences of sexting behavior when it exists in the context of a consensual interaction. As speculated by leading researchers in this field, sexting may represent a normal extension of sexual development for technologically-immersed adolescents and emerging adults [7,15,52,56], and researchers have even advocated for “safe sexting” educational curricula [57]. This “normalcy discourse” [9] may suggest that sexting is a healthy expression of sexual desire and exploration of sexual desire among emerging adults. On the other end of the spectrum, sexting that is unwanted or coerced could represent an extension of IPA and sexual violence. If we are to better understand the nature of sexting, including the potential benefits and risks of sexting, it is critical to differentiate sexting that occurs in a normative, consensual context from that which does not, by examining it on its own. It is also imperative that we examine how an individual’s historical experiences, including IPA, affect an array of consequential outcomes of sexting, even when it is considered by the sexter to be consensual.

Therefore, the current project was designed to comprehensively examine a range of potential positive and negative consequences of sexting in the context of a consensual sexting episode. Because the data examining the consequences of sexting is limited, particularly related to positive outcomes, we sought to systematically and comprehensively examine those consequences within existing behavior analytic models of human behavior. Further, because it is possible that certain life experiences or circumstances, including history of IPA victimization, may contribute to unique vulnerabilities related to sexting, this research also examined how an individual’s victimization history differentially impacted the context and consequences of consensual sexting.

Based on the extant literature, we hypothesized:

**Hypothesis 1 (H1).**  *Emerging adults, both men and women, would report that, despite labeling their sexting experience as consensual, they experienced coercion and pressure to sext.*

**Hypothesis 2 (H2).**  *Significant positive associations would occur between sexting coercion and all forms of IPA.*

**Hypothesis 3 (H3).**  *Individuals reporting IPA victimization, as compared to those who did not report IPA victimization, would report different consequences from their sexting experiences, including higher rates of overall punishing consequences, as well as negative/positive punishment (H3a), and lower rates of overall reinforcement, both positive and negative (H3b).*

## 2. Methods

### 2.1. Participants

Undergraduate students (*N* = 536; 77.2% female) were recruited from psychology courses at a large, public, Midwestern university to participate in the present study. To be eligible, participants were required to be at least 18 years of age at the time of the study and report that they had “sent a consensual sext (a sexually explicit picture or video through any technological medium)” in their adult life. Participants’ mean age was 19.00 years (*SD* = 1.67; range = 18–36), and most identified as heterosexual (86.6%). Consistent with the demographics of the university, most students identified as Caucasian (87.9%), with 7.3% of the sample identifying as Hispanic/Latino. Academically, most students were freshmen (67.1%), followed by sophomores (20.1%), juniors (8.6%), seniors (3.4%), and one person identifying as “Other”. The majority of participants (59.3%) reported currently being involved in a dating or intimate relationship.

### 2.2. Procedure

All procedures and methods were reviewed and approved by the research ethics board at the university associated with the first author. Participants were recruited between February 2019 and March 2020 through an introductory psychology research portal that provided a brief description of the study and the eligibility requirements, and participants received class credit for signing up and participating in the study. Interested and eligible participants were provided with a link to an online survey website (e.g., Qualtrics) that used encryption to maintain participant confidentiality. The survey was designed to be completely anonymous, IP address collection was disabled, and no personally identifying information was collected. Following completion of the online informed consent documents, participants were provided with a series of measures to complete. At the conclusion of the study, participants were provided with the name, email address, and phone number of the first author in the event that they experienced distress resulting from the research. They were also provided with a list of local and campus referrals for domestic violence and counseling services.

### 2.3. Measures

Demographics. Participants indicated their age, academic standing, dating, sexual, and sexting history, ethnicity, gender, and sexual orientation.

Sexting Experiences. Using an open-response format, participants reported the number of different people to whom they had ever sent a sext (defined as “a sexually explicit picture or video through any technological medium (text message, Snapchat, Instagram, Messenger, or any other application) the number of different people from whom they had received a sext, the estimated lifetime prevalence of sexts sent and received, and the age at which they had first sent and received sexts.

Sexting Context. In order to direct participants to think about a specific instance and relationship context in which they had sent a consensual sext, participants were instructed to “Think about the most recent person with whom you sent a consensual sext (sexually explicit picture or video message) through any technological medium.” They were asked to describe the degree to which the person was known to them (not well, a little, fairly well, very well), how long they knew them, the gender of the person with whom they sexted, the nature of the sext (sexually suggestive but clothed, nearly nude, nude, picture or video of sexual act or masturbation), and the age at which they sexted this person. They also indicated the type of relationship in which this sexting occurred: friends with benefits, one-night stand, fuck buddy, booty call, cheating partner, committed partner, someone they knew only online, or a casual dating partner.

Sexting Consequences. To measure the consequences of consensual sexting, an in-depth questionnaire was designed to gather comprehensive information on the context and dynamics surrounding the specific episode of consensual sexting. The questionnaire was conceptualized from a functional analytic perspective and was modified from existing interviews assessing contextual variables in intimate partner violence [49,58]. To provide an inclusive and widespread analysis of possible consequences framed within behavioral theory, a range of various possible outcomes and their potential operant functions were included. This included questions related to a range of potential positive reinforcers resulting from the sext, both at the individual and relationship level (e.g., “After you sent the sext, did your partner seem more emotionally connected to you?”). This subscale included 24 items. Nine items were included that captured possible individual and relational negative reinforcing consequences, reflecting avoidance or escape of aversive outcomes that might increase the likelihood of sexting in the future (e.g., “Did you feel less lonely after sending the sext?”). A total reinforcement subscale was created by summing scores from the positive and negative reinforcement subscales. Finally, we included a range of potential punishing consequences, which presumably would decrease future sexting activity. These included items such as “Did you feel ashamed after sending the text?” and “After you sent the sext to your partner, did you find that you were less satisfied with your relationship with your partner?”, again, assessing potential punishers at the individual and relationship level. Though these 28 items were conceptualized to capture both negative and positive punishers, for ease of interpretation they were collapsed into one punishment subscale. The internal consistency of all subscales was good (positive reinforcement, α = 0.940; negative reinforcement, α = 0.830; reinforcement, α = 0.945; punishment, α = 0.932). The items that comprised this measure are listed in Appendix A, Table A1 and Table A2.

IPA. The Revised Conflict Tactics Scale (CTS2) [59] is one of the most commonly utilized measures examining IPA and has demonstrated good reliability and validity in prior research. This 78-item measure assesses physical, sexual, and psychological aggression that has occurred in the past year. Participants indicated how frequently they had perpetrated and been victimized by each behavior on a 7-point scale ranging from 0 (this never happened) to 6 (more than 20 times). Variety scores were calculated [60] to provide count data on perpetration and victimization of each form of IPA in the past year. Dichotomous variables were also created to reflect the occurrence or non-occurrence of perpetration and victimization for each type of IPA in the past year. In the present study, only the victimization subscales were utilized. Consistent with prior literature, internal consistency estimates of the variety scores for each subscale were adequate (psychological victimization α = 0.745; physical victimization α = 0.885; sexual victimization α = 0.660).

Sexting Coercion. A modified version of the 34-item Sexual Coercion in Intimate Relationships Scale (SCIRS_S) [61] was utilized to measure sexting coercion in their current or most recent intimate relationship. The measure differentiates between different types of coercive acts in sexting and has been used in other research to assess sexting coercion [17]. Participants were presented with a series of potentially coercive acts (e.g., “My partner persisted in asking me to send them a sexually explicit picture or video, even though I did not want to”) and asked to rate this on a 6-point scale ranging from 0 (act has not occurred) to 5 (act occurred 11+ times). This measure includes a total score, as well as subscales that examine coercive acts in which gifts or benefits are withheld or threatened to be withheld (Resource Manipulation: RM), coercive acts related to the couple’s relationship and access to sexting (Commitment Manipulation: CM), and threats to pursue relationships with other people if sexting does not occur (Defection Threat: DT). Cronbach’s alpha for this measure was 0.910 for the total score, and good for all subscales (RM α = 0.928; CM α = 0.924; DT α = 0.945).

## 3. Results

### 3.1. Sexting Experiences and Gender

All participants included in the sample reported a history of sending at least one sext and 94% of the sample also reported a history of receiving a sext. Within the entire sample, the average number of sexts sent to another person was 33.15 (*SD* = 38.06) and the average number of sexts received from another person was 43.96 (*SD* = 47.93). The average age of sending a first sext was 16.57 years old (*SD* = 1.69) and the average of receiving a first sext 16.15 years old (*SD* = 6.94). On average, participants reported receiving sexts from approximately six different people (*M* = 6.12; *SD* = 5.89) and sending sexts to about four different people (*M* = 3.69; *SD* = 3.33). Within their most recent consensual sexting relationship in which they sent a sext, the average number of sexts sent was 41.28 (*SD* = 108.43) and the average number of sexts received was 36.94 (*SD* = 102.22). In terms of relationship context, 52.4% of the sample stated that their sext was sent to a casual dating partner, whereas 47.6% reporting sexting in the context of a committed relationship. Table 1 provides a summary of sexting consequences for the entire sample.

Men and women did not differ significantly in number of sexts sent and received, age of first sexting activities, and number of people engaged with in sexting activities. Table 1 provides a summary of gender differences in sexting consequences. Women reported experiencing significantly more punishing consequences after sexting (*M* = 6.08, *SD* = 6.52) than men reported (*M* = 4.58, *SD* = 5.54), *t*(229.34) = −2.52, *p* = 0.01. No other significant gender differences in sexting consequences were identified.

### 3.2. Relationship between Sexting Experiences and IPA

Approximately 57% of the entire sample reported a history of psychological IPA victimization in the past year. Additionally, 17.2% of the sample endorsed a past year history of physical IPA victimization and nearly 32% of the sample reported experiencing sexual IPA victimization in the past year. The average number of IPA acts experienced in the past year was 1.41 (*SD* = 1.47) for psychological IPA victimization, 0.49 (*SD* = 1.32) for physical IPA victimization, and 0.61 (*SD* = 1.00) for sexual IPA victimization. Men and women did not differ significantly in their reporting of victimization experiences for any IPA form.

Individuals with a history of psychological IPA victimization reported being younger when receiving (*M* = 15.75, *SD* = 1.78) and sending (*M* = 16.38, *SD* = 1.69) their first sexts relative to those without a psychological IPA victimization history (*M* = 17.15, *SD* = 11.74; *M* = 16.96, *SD* = 1.65), *t*(451) = 1.98, *p* = 0.048 and *t*(479) = 3.68, *p* = 0.00, respectively. Participants with a sexual IPA victimization history also reported being younger when sending their first sexts (*M* = 16.26, *SD* = 1.68) relative to those without a sexual IPA victimization history (*M* = 16.77, *SD* = 1.68), *t*(479) = 3.19, *p* = 0.00. Additionally, those with a sexual IPA victimization history reported sending (*M* = 4.43, *SD* = 3.52) and receiving (*M* = 7.28, *SD* = 6.16) sexts with a larger number of people than those without a sexual IPA victimization history (*M* = 3.08, *SD* = 2.98; *M* = 5.17, *SD* = 5.45), *t*(298.43) = −4.22, *p* = 0.00 and *t*(297.47) = −3.62, *p* = 0.00, respectively. Those with and without a history of physical IPA victimization did not differ in number of sexts sent and received, age of first sexting activities, or number of people engaged with in sexting activities.

### 3.3. Relationships between Sexting Coercion and IPA

Approximately half of the sample (49.2%) reported consenting to send sexts when they actually did not want to, on at least some occasions. Nearly 28% of the sample said they felt at least a little coerced about sending the first sext in their most recent consensual sexting relationship, with 41.8% of participants stating that they felt some pressure to send the first sext. Beyond the first sext, 22% of the sample said that they felt pressured or coerced to send sexts on other occasions. Table 1 provides a summary of means and standard deviations for the entire sample’s total SCIRS_S scores and each of the SCIRS_S subscale scores, along with comparisons between men and women on SCIRS_S total and subscale scores. No gender differences in SCIRS_S scores were identified in the sample. Men and women did not differ significantly in how often they consented to sending a sext when they did not want to nor the extent to which they felt pressured or coerced when sending the first sext in their most recent consensual sexting relationship.

Those with a physical IPA victimization history were more likely to report feeling coerced to send the first sext in their most recent sexting relationship (*M* = 1.60, *SD* = 0.94) than those without a physical IPA victimization history (*M* = 1.37, *SD* = 0.75), *t*(120.10) = −2.18, *p* = 0.03, and those with a sexual IPA victimization history reported greater pressure to send the first sext in their most recent sexting relationship (*M* = 1.58, *SD* = 0.94) relative to those without a sexual IPA victimization history (*M* = 1.76, *SD* = 0.91), *t*(353.70) = −1.99, *p* = 0.048. As reflected in Table 2, SCIRS_S total and subscale scores were positively correlated with all forms of IPA victimization. Table 3, Table 4 and Table 5 summarize comparisons in SCIRS_S total and subscale scores between those with and without IPA victimization histories. Those with a physical or sexual IPA victimization history, relative to those without a physical or sexual IPA victimization history, had higher SCIRS_S total and all SCIRS_S subscale scores. People with a psychological IPA victimization history reported higher SCIRS_S total, SCIRS_S-RM, and SCIRS_S-CM scores than those without a psychological IPA victimization history.

### 3.4. Relationships between Sexting Consequences and IPA

Table 3, Table 4 and Table 5 summarize differences in sexting consequences based on IPA victimization history. Across all three forms of IPA victimization, those with an IPA victimization history reported experiencing more negative reinforcing consequences after sending a sext than those without an IPA victimization history. Those reporting a physical and sexual IPA victimization history also reported experiencing more punishing consequences after sending a sext compared to those without a physical or sexual IPA victimization history. Compared to those without a sexual IPA victimization history, those with a history of sexual IPA victimization reported greater overall reinforcing consequences after sending a sext.

## 4. Discussion

As electronic sexual communication has become more commonplace, researchers have explored the positive and negative correlates and outcomes of sexting. Overall, these studies have shown that sexting more frequently in relationships is related to sexual satisfaction [42], and in some cases, relationship satisfaction [40,43,44,45]; however, sexting is also related to myriad of negative correlates, including conflict, ambivalence, and insecure attachment patterns [42]. Moreover, and more relevant to the current inquiry, sexting, especially unwanted or coerced sexting, has been found to relate to negative psychological symptoms, dating violence, offline sexual coercion, and IPA e.g., [34,35,42,54].

At the same time, a separate line of research has examined motivations for and outcomes of sexting, finding that most individuals have positive expectations regarding the outcome of sexting and do so for fun, flirtation, or to initiate sex [10,20]. Additionally, most individuals report more positive than negative immediate outcomes of sexting [14,46]. Hence, a duality exists whereby the motivations, expectations, and immediate outcomes for sexting are reported to be mostly positive, but relationship correlates are often negative. In this study, we combined and expanded these lines of inquiry to examine the extent to which IPA is associated with coercive sexting experiences and also whether those who experience IPA, compared to those without a recent IPA history, differ in their experiences of positive and negative consequences of sexting. Finally, unlike previous studies, we specified examined coerced sexting and positive and negative consequences within the context of sexting experiences that participants deemed consensual.

In our sample of sexters, sexting was common—young adults reported sending an average of 41 sexual pictures or videos to their last relationship partner alone. Additionally, we found that both IPA and coercive sexting were common in our sample, with more than half of participants (57%) reporting some type of IPA history (psychological IPA was most common), almost half (49%) reporting that they had felt coerced to sext, and one quarter (28%) reporting that they felt pressured into sending the first sext in their last consensual sexting experience. The high rates of both IPA and sexting coercion were not unexpected; they align with previous research on both the prevalence of IPA and sexting coercion [17,38]. The presence of pressure and coercion within sexting episodes characterized as consensual was also expected (supporting our H1), and this finding gives some context to the previous literature on coercive and unwanted sexting [38,53]. Namely, it suggests that at least some of the coercive sexual experiences, and perhaps even some deemed traumatic to these participants, were considered consensual acts. Unexpectedly, gender differences did not emerge with regard to IPA or sexting coercion, which was somewhat inconsistent with previous research. For example, recent data found that women were 2.49 times more likely to be pressured to sext, and 5.06 times to be threatened related to sexting [55]. Our data did not suggest such gender differences, perhaps due to measurement and methodological differences. From a research perspective, this finding suggests that more delineation and nuance is warranted in investigations of unwanted and coerced sexting, exploring the ways in which consent, coercion, and unwanted sexual behavior manifest across sexting episodes and/or different relationship types. Meanwhile, in terms of practical implications, these findings suggest that more discussion and education is necessary regarding the definition of sexual coercion and the negotiation of consent within online sexual experiences, as it is a complicated process [7]. Clearly, teens and young adults need to be equipped with communication and negotiation strategies to help them recognize and resist sexual coercion online, particularly because sexting episodes that are deemed coercive are likely to cause individuals (especially women) trauma both at the time they happen and in future, when the coerced party looks back on the event [38]. This could perhaps be embedded within sexual education in the form of “safe sexting” lessons [57] or even in health classes as part of a module on healthy relationship communication patterns and sexual harassment.

Regarding our second hypothesis, as predicted, the experience of all types of IPA victimization (psychological, physical, and sexual) in the last year was significantly related to all subscales of coercive sexting in the current or most recent relationship. Moreover, those who experienced these different forms of IPA were significantly more likely than those who did not experience these forms of IPA to report nearly every type of sexting coercion (with the exception that was there was no difference in experiences of defection threat among those who did and did not experience psychological IPA). Our findings support previous research suggesting that coerced sexting is related to partner victimization [34,35,42,54,55] and again reinforce the idea proposed by Ross et al. [17] that sexting coercion be considered part of the construct of IPA. As partner victimization and coercive control extend to digital formats, it will be important to reconceptualize common definitions of IPA and sexual harassment to include digital violence. Additionally, it may be important for advocates to provide education to individuals that digital mechanisms might be used to perpetrate different types of partner aggression, including stalking, sexual aggression, sexual harassment, physical aggression (in the form of threats), and psychological aggression.

Hypothesis three, related to the different consequences of sexting based on history of IPA victimization, was partly supported. Those who reported physical and sexual IPA victimization perceived more punishing consequences overall, as anticipated. These findings were particularly salient for females, as they were more likely to identify punishing consequences, compared to males. These findings provide an additional context for examining prevailing data with regard to gender differences in sexting. While some research has noted that males and females engage in sexting at similar rates [14,16], it appears that the experience of sexting may differ across gender, further necessitating a more nuanced approach to prevention and education. These data suggest that despite commonly identified motivations of fun, flirtation, and relationship satisfaction, particularly for those with a history of physical or sexual victimization, these motivations may not be the reality of their experiences. Rather, it appears that they are more likely to experience undesirable outcomes of consensual sexting, including regret, embarrassment, trauma, and shame, compared to their non-victimized counterparts. Additionally, recent research examined the interrelationship of sexting and online sexual victimization, and found that for females specifically, these experiences were correlated with poorer mental health [55]. These negative experiences are similar to the undesirable consequences reported by individuals who engage in unwanted sexting [54] and suggest that sexting coercion, unwanted sexting, and the experience of IPA are intertwined in complex ways.

Interestingly, those who had experienced any form of IPA victimization (psychological, physical, or sexual) reported significantly more negatively reinforcing sexting experiences than those who had not experienced those forms of IPA. With regard to the negative reinforcement effects, this finding aligns somewhat with Drouin and Tobin’s [53] findings that some women (those who were anxiously attached to their partners) were more likely to engage in unwanted sexting because they wanted to avoid an argument with their partner. In both the current research and this previous study [55], the removal of an unpleasant stimulus served to increase a sexting behavior that was either unwanted or considered coercive. Although removal of an unpleasant outcome is not inherently bad from a behavioral perspective, those who are pressured into sexting to avoid an argument or discord with a partner may be more likely to experience regret or trauma, especially if the sexting was considered coerced [38]. This finding has important implications for prevention and education efforts, as it further emphasizes the need to explore issues of coercion and pressure in sexting, and ensure that if a person chooses to engage in the sharing of sexual images, this is done for relationship and individual enhancement (i.e., positive reinforcement), rather than to alleviate guilt or partner pressure.

Finally, our finding that those who experience sexual IPA victimization are also significantly more likely than those who do not experience sexual IPA to have positive sexting consequences overall highlight the dualistic nature of sexting, in general: it is motivated by both positive (to some) and negative (to some) feelings, and can have both positive and negative consequences. However, it is important to note that those who experience sexual IPA victimization appear to have more of this duality in their experiences, which poses a clinical challenge in that it may be difficult to make individuals cognizant of the punishments from sexting if their behaviors are also being reinforced in some way.

### Limitations and Future Directions

The results of this study provide an important contribution to the understanding of consent and perceived consequences (both positive and negative) related to sexting. However, these results should be interpreted in light of their limitations. First, the relative homogeneity of our sample may limit the generalizability of our results. All participants were college-attending and a majority identified as heterosexual and/or Caucasian, which prevented any analysis on how the nature of consent and consequences of sexting may vary as a function of race, culture, or sexuality. Some research has determined that online and long-distance dating may be more prevalent in non-heterosexual relationships; however, the impact of sexting in these relationships has yet to be explored [62,63]. Additionally, this sample was recruited from a psychology course research pool that was predominately composed of female students, further limiting the generalizability of our results and our ability to detect gender differences in sexting, sexting coercion, and IPA. Second, the cross-sectional nature of this study renders analysis of temporal relationships between variables and causal hypotheses impossible. Specifically, we were unable to assess whether a causal relationship between experience of IPA and perceived consequences of sexting exists. Third, all variables were collected via self-report measures which introduced the potential for socially desirable responding. Studies have indicated that sexting is relatively stigmatic in our current social and legal environment, with some even referring to sexting as a potential deviant behavior [64,65]. It is possible that participants may be less likely to report some sexting behaviors due to the high level of stigma they may experience. Future studies should statistically control for potential socially desirable responding when possible. Finally, results should be interpreted in light of the limitations of the measures employed in this research. The use of a measure of sexting consequence that has not been validated in this population is a limitation, and future research on the psychometrics of this measure as well as with other populations is necessary. Additionally, while the CTS2 is widely used within the field of IPA research, this is a behaviorally specific measure that fails to adequately address motivation, context, or consequences of IPA. The internal consistency of CTS2 items measuring sexual coercion has been found to be weaker than the internal consistency of the CTS2 items assessing psychological and physical IPA [66], which may have impacted the current study’s findings. Within the current study, the internal consistency of the CTS2 sexual coercion subscale was lower than the other CTS2 subscales, with the internal consistency of this subscale being particularly low for men (α = 0.46), relative to women (α = 0.68). Poor internal consistency for the CTS2 sexual coercion subscale could have impacted detection of true but unknown gender differences in sexual IPA victimization rates. Future research in this field should consider the addition of other measures to more broadly assess forms of IPA, especially sexual IPA victimization, and determine contextually-specific features of IPA and sexting coercion.

While our data provide an initial examination of the perceived consequences for consensual sexting, future research is necessary to continue to understand the nuances of coercion, IPA, contextual control, and gender in sexting scenarios for emerging adults. In the current sample, there were no differences between men and women on rates of IPA, including sexual victimization and sexting coercion. While some previous research has found similar rates of sexual victimization in dating samples e.g., [67], future research should further elucidate whether these findings endure in samples with higher rates of gendered sexual victimization. Additionally, future research should consider comprehensive examination of perceived consequences across relationship type, including casual sexual relationships, and the interrelationships with IPA. While this inquiry was outside the scope of the current project, other research has demonstrated differences in consequences across relationship type [14], and integration of these methods would improve our understanding of coerced sexting in a range of contexts. Finally, this research should be examined in light of existing research on sexual harassment, both in terms of the overlap between this construct and sexting coercion and IPA, and to determine how sexual harassment differs from sexting coercion in self-identified consensual contexts. This research would greatly expand the complexity of our understanding of sexting in the range of contexts, situations, and relationships in which it occurs, and lead to more informed education and prevention efforts.

In summary, critical to our inquiry was the use of a behavioral framework to categorize sexting consequences. To our knowledge, this is the first time this framework has been applied to sexting behavior; however, the use of this framework allowed us to delineate sexting consequences in ways that may help us better understand why individuals engage in sexting within different relationship contexts. Overall, our findings suggest that even in the context of consensual sexting, coercion and pressure is often present, particularly for those who have experienced coercion in other contexts. Our data further emphasize the interrelationship between IPA and sexting coercion, which has important implications for the conceptualization of aggression that occurs in relationships, as well as educational efforts. Our data suggest that while individuals with a history of some forms of IPA victimization report more punishing consequences, they also report reinforcing consequences, particularly negative reinforcement. These data provide a critical context in which to recognize the complexity of sexting and sexting coercion, and the potential outcomes of such sexting for some individuals. While behaviors maintained via negative reinforcement are not inherently problematic, it is worth considering, and educating, individuals on whether their sexting is rooted in achieving desirable outcomes or alleviating unpleasant emotional or relationship states. Particularly in light of recent conceptualizations of sexting as a developmentally normative behavior, it is worth challenging adolescents and emerging adults as to the reasons and motivations for sexting, to ensure that such behavior is not grounded in problematic interpersonal and intrapersonal processes.

## Figures and Tables

**Table 1:** Gender differences in sexting consequences and sexual coercion.

Variable	Full Sample	Men	Women		
Consequences	*n* = 534 ^†^	*n* = 122 ^†^	*n* = 412 ^†^		
	M (SD)	M (SD)	M (SD)	*t*(df)	*d*
Reinforcing Consequences	16.24 (0.17)	15.02 (9.23)	16.60 (9.14)	−1.67 (532)	0.17
Positive Reinforcement	16.50 (5.33)	15.66 (5.39)	16.74 (5.30)	−1.70 (405)	0.20
Negative Reinforcement	3.66 (2.67)	3.47 (2.55)	3.72 (2.71)	−0.91 (532)	0.10
Punishing Consequences	5.74 (6.34)	4.58 (5.54)	6.08 (6.52)	−2.52 (229.34) *	0.25
SCIRS	*n* = 489	*n* = 105	*n* = 384		
	M (SD)	M (SD)	M (SD)	*t*(df)	*d*
SCIRS-Total	2.21 (6.45)	1.81 (5.21)	2.32 (6.75)	−0.72 (487)	0.08
SCIRS-RM	0.53 (2.18)	0.53 (2.14)	0.53 (2.19)	0.03 (487)	0.00
SCIRS-CM	1.46 (4.31)	1.17 (3.38)	1.53 (4.53)	−0.76 (487)	0.09
SCIRS-DT	0.23 (1.50)	0.10 (0.62)	0.26 (1.66)	−0.96 (487)	0.13

Note: SCIRS = Sexual Coercion in Intimate Relationships Scale-Sexting Version; SCIRS-RM = SCIRS-Resource Manipulation/Violence; SCIRS-CM = SCIRS-Commitment Manipulation; SCIRS-DT = SCIRS-Defection Threat. † Sample sizes were smaller for positive reinforcement, with 407 people responding, including 90 men and 317 women. * *p* < 0.05.

**Table 2:** Correlations between IPA Victimization and Sexting Coercion.

Variables	1.	2.	3.	4.	5.	6.	7.
1. Psychological IPA Victimization	---						
2. Physical IPA Victimization	0.47 *** (*n* = 482)	---					
3. Sexual IPA Victimization	0.38 *** (*n* = 484)	0.40 *** (*n* = 482)	---				
4. SCIRS_S-Total	0.27 *** (*n* = 481)	0.30 *** (*n* = 480)	0.48 *** (*n* = 481)	---			
5. SCIRS_S-RM	0.20 *** (*n* = 481)	0.31 *** (*n* = 480)	0.39 *** (*n* = 481)	0.81 *** (*n* = 489)	---		
6. SCIRS_S-CM	0.26 *** (*n* = 481)	0.21 *** (*n* = 480)	0.41 *** (*n* = 481)	0.91 *** (*n* = 489)	0.58 *** (*n* = 489)	---	
7. SCIRS_S-DT	0.12 ** (*n* = 481)	0.23 *** (*n* = 480)	0.31 *** (*n* = 481)	0.50 *** (*n* = 489)	0.38 *** (*n* = 489)	0.21 *** (*n* = 489)	---

Note: IPA = Intimate Partner Aggression; SCIRS_S = Sexual Coercion in Intimate Relationships Scale–Sexting Version; SCIRS_S-RM = SCIRS_S-Resource Manipulation/Violence; SCIRS_S-CM = SCIRS_S-Commitment Manipulation; SCIRS_S-DT = SCIRS-S-Defection Threat. ** *p* < 0.01; *** *p* < 0.001.

**Table 3:** Differences in Sexting Consequences and Sexual Coercion Based on Psychological IPA Victimization History.

Variable	No Psychological IPA Victimization	Psychological IPA Victimization		
Consequences	*n* = 177 ^†^	*n* = 307 ^†^		
	M (SD)	M (SD)	*t*(df)	*d*
Reinforcing Consequences	16.31 (8.98)	16.54 (9.42)	−0.27 (482)	0.02
Positive Reinforcement	16.62 (5.42)	16.74 (5.33)	−0.21 (369)	0.02
Negative Reinforcement	3.16 (2.70)	3.94 (2.69)	−3.09 (482) **	0.29
Punishing Consequences	4.85 (5.94)	5.87 (6.41)	−1.73 (482)	0.17
SCIRS_S	*n* = 175	*n* = 306		
	M (SD)	M (SD)	*t*(df)	*d*
SCIRS_S-Total	0.65 (2.86)	3.16 (7.71)	−5.12 (426.43) ***	0.43
SCIRS_S-RM	0.11 (0.84)	0.78 (2.65)	−4.09 (399.96) ***	0.34
SCIRS_S-CM	0.39 (1.36)	2.10 (5.24)	−5.38 (372.31) ***	0.45
SCIRS_S-DT	0.15 (1.19)	0.28 (1.67)	−0.93 (479)	0.09

Note: IPA = Intimate Partner Aggression; SCIRS_S = Sexual Coercion in Intimate Relationships Scale–Sexting Version; SCIRS_S-RM = SCIRS_S-Resource Manipulation/Violence; SCIRS_S-CM = SCIRS_S-Commitment Manipulation; SCIRS_S-DT = SCIRS_S-Defection Threat. † Sample sizes were smaller for positive reinforcement, with 140 people with no psychological IPA victimization history and 231 people with a psychological IPA victimization history reporting. ** *p* < 0.01; *** *p* < 0.001.

**Table 4:** Differences in Sexting Consequences and Sexual Coercion Based on Physical IPA Victimization History.

Variable	No Physical IPA Victimization	Physical IPA Victimization		
Consequences	*n* = 390 ^†^	*n* = 92 ^†^		
	M (SD)	M (SD)	*t*(df)	*d*
Reinforcing Consequences	16.23 (9.28)	17.55 (9.01)	−1.24 (480)	0.14
Positive Reinforcement	16.59 (5.43)	17.38 (4.68)	−1.11 (367)	0.16
Negative Reinforcement	3.41 (2.67)	4.71 (2.70)	−4.16 (480) ***	0.48
Punishing Consequences	5.19 (5.87)	6.90 (7.61)	−2.03 (117.80) *	0.25
SCIRS-S	*n* = 388	*n* = 92		
	M (SD)	M (SD)	*t*(df)	*d*
SCIRS_S-Total	1.21 (3.80)	6.64 (11.71)	−4.39 (95.60) ***	0.62
SCIRS_S-RM	0.22 (1.17)	1.87 (4.18)	−3.75 (94.41) ***	0.54
SCIRS_S-CM	0.93 (3.02)	3.80 (7.33)	−3.69 (98.46) ***	0.51
SCIRS-S-DT	0.06 (0.44)	0.97 (3.25)	−2.68 (91.80) ***	0.39

Note: IPA = Intimate Partner Aggression; SCIRS_S = Sexual Coercion in Intimate Relationships Scale–Sexting Version; SCIRS_S-RM = SCIRS_S-Resource Manipulation/Violence; SCIRS_S-CM = SCIRS_S-Commitment Manipulation; SCIRS_S-DT = SCIRS_S-Defection Threat. † Sample sizes were smaller for positive reinforcement, with 301 people with no physical IPA victimization history and 68 people with a physical IPA victimization history reporting. * *p* < 0.05; *** *p* < 0.001.

**Table 5:** Differences in Sexting Consequences and Sexual Coercion Based on Sexual IPA Victimization History.

Variable	No Sexual IPA Victimization	Sexual IPA Victimization		
Consequences	*n* = 390 ^†^	*n* = 92 ^†^		
	M (SD)	M (SD)	*t*(df)	*d*
Reinforcing Consequences	15.69 (9.36)	17.89 (8.89)	−2.51 (482) *	0.24
Positive Reinforcement	16.32 (5.68)	17.38 (4.66)	−1.94 (311.19)	0.20
Negative Reinforcement	3.20 (2.67)	4.51 (2.60)	−5.22 (482) ***	0.50
Punishing Consequences	4.85 (5.81)	6.69 (6.87)	−2.96 (298.21) **	0.29
SCIRS_S	n = 388	n = 92		
	M (SD)	M (SD)	*t*(df)	*d*
SCIRS_S-Total	0.78 (3.29)	4.98 (9.46)	−5.57 (188.95) ***	0.59
SCIRS_S-RM	0.22 (1.63)	1.12 (2.90)	−3.70 (224.83) ***	0.38
SCIRS_S-CM	0.50 (2.11)	3.31 (6.37)	−5.56 (186.83) ***	0.59
SCIRS_S-DT	0.06 (0.44)	0.55 (2.46)	−2.58 (172.71) *	0.28

Note: IPA = Intimate Partner Aggression; SCIRS_S = Sexual Coercion in Intimate Relationships Scale–Sexting Version; SCIRS_S-RM = SCIRS_S-Resource Manipulation/Violence; SCIRS_S-CM = SCIRS_S-Commitment Manipulation; SCIRS_S-DT = SCIRS_S-Defection Threat. † Sample sizes were smaller for positive reinforcement, with 241 people with no sexual IPA victimization history and 130 people with a sexual IPA victimization history reporting. * *p* < 0.05; ** *p* < 0.01; *** *p* < 0.001.

## Appendix A

**Table A1:** Reinforcement Subscale.

1	After you sent the sext, did your partner seem more emotionally connected to you?	+
2	After you sent the sext, did your partner report more sexual arousal toward you?	+
3	Did you feel like you fulfilled your partner’s needs after you sent the sext?	+
4	Did you feel more powerful in the relationship after you sent the sext?	+
5	Did your partner reciprocate with a sext after you sent your sext?	+
6	Do you think that your partner paid closer attention to you after you sent the sext?	+
7	Do you think the sext made your partner more committed to you?	+
8	Do you think the sext made you more committed to your partner?	+
9	Do you think the sext increased the trust that you had in that person?	+
10	Do you think the sext increased your investment in the relationship?	+
11	Do you think the sext increased your partner’s investment in the relationship?	+
12	Do you think the sext increased the intimacy of the relationship?	+
13	Do you think the sext made the relationship more interesting?	+
14	Do you think the sext increased the exclusivity (that you or your partner would not date other people) of your relationship?	+
15	Did you feel more satisfied with your relationship after you sent the sext?	+
16	Did you feel more flirtatious toward your partner after you sent the sext?	+
17	Did you feel more emotionally connected with your partner after you sent the sext?	+
18	Did you feel more physically aroused (e.g., feel more energized, excited) after you sent the sext?	+
19	Did you feel more sexually aroused after you sent the sext?	+
20	Did you feel more attractive/sexy/desired after you sent the sext?	+
21	Did you feel better about yourself after you sent the sext?	+
22	After you sent the sext, did your partner praise you?	+
23	After you sent the sext, did anyone else (friends, peers, etc.) praise you?	+
24	After you sent the sext, did you feel like you fit in better with your friends/peers?	+
25	Do you think that sexting your partner prevented an argument/disagreement?	
26	Do you think sexting your partner prevented him/her from terminating the relationship?	
27	Did you feel LESS emotionally upset or insecure about your relationship?	
28	Did you feel LESS physically tense?	
29	Did you feel LESS guilty?	
30	Did you feel LESS bored after sending the sext?	
31	Did you feel LESS lonely after sending the sext?	
32	Did you feel LESS worry about the stability of your relationship?	
33	Did you feel LESS pressure to sext after you sent the sext?	

+ indicates that it loaded on the positive reinforcement scale.

**Table A2:** Punishment Subscale.

1	Did you experience regret immediately after sending the sext?	+
2	Did you experience regret at a later point after sending the sext?	+
3	Was the sext distributed to others outside the person you sent it to?	+
4	Immediately after, did you experience worry that the sext would be distributed to others outside of the person you sent it to?	+
5	Did you worry at a later point that the sext would be distributed to others outside of the person that you sent it to?	+
6	Did your partner tell other people that you sent a sext?	+
7	Immediately after, did you experience worry about how people would react if they knew you had sent a sext?	+
8	Did you worry at a later point about how people would react if they knew you had sent a sext?	+
9	Immediately after sending the sext, were you upset or traumatized?	+
10	At a later time, were you upset or traumatized by sending the sext?	+
11	Did you feel awkward/embarrassed after sending the sext?	+
12	Did you feel vulnerable after sending the sext?	+
13	Did you feel desperate after sending the sext?	+
14	After you sent the sext, did you feel disgusted with yourself/your behavior?	+
15	After you sent the sext, did you feel disappointed in yourself/your behavior?	+
16	After you sent the sext, did you feel ashamed?	+
17	Did your partner criticize, scold, or condemn you for sending the sext?	+
18	After the incident, did someone else criticize, scold, or condemn you for sending the sext?	+
19	After you sent the sext, did your partner terminate the relationship?	+
20	Did your partner seem more emotionally upset/distressed (e.g., more disappointed, frustrated, angry, sad, jealous, scared, etc.) after you sent the sext to him/her?	+
21	Did you feel ashamed after sending the sext?	+
22	After you sent the sext, did you find that relationship tension between you and your partner increased?	+
23	Did you feel more emotionally upset/distressed (e.g., more disappointed, frustrated, sad, jealous, scared, etc.) after you sent the sext to your partner?	+
24	After you sent the sext to your partner, did you find that you were LESS satisfied with your relationship with your partner?	
25	After you sent the sext to your partner, did you find that your partner was LESS satisfied with the relationship?	
26	Did you feel LESS emotionally connected to your partner after you sent the sext?	
27	Did you feel LESS sexually connected to your partner after you sent the sext?	
28	Did it reduce your interest in engaging in in-person sexual behaviors (kissing, sexual touching, oral, anal, and/or vaginal sex) with this person?	

+ indicates that it was originally conceptualized as positive punishment.
